# ﻿New arthropod-Podostemaceae associations in Central and South America

**DOI:** 10.3897/zookeys.1129.91398

**Published:** 2022-11-11

**Authors:** Jacob Bethin, Rayda K. Krell, C. Thomas Philbrick

**Affiliations:** 1 University of Florida, Entomology and Nematology Department, 1881 Natural Area Dr., Gainesville, FL 32608, USA University of Florida Gainesville United States of America; 2 Western Connecticut State University, Department of Biology, 181 White St., Danbury, CT 06810, USA Western Connecticut State University Danbury United States of America

**Keywords:** Aquatic arthropods, freshwater biodiversity, lotic invertebrates, plant-insect interactions, river ecosystems

## Abstract

Podostemaceae are a unique family of aquatic angiosperms found in river rapids and waterfalls throughout southern Asia, Africa, and the Americas. Podostemaceae are understudied, and consequently, the arthropods associated with these plants are not well known. We sought to expand knowledge of arthropod-Podostemaceae associations to better understand the impact of these plants on aquatic ecosystems and biodiversity. We examined samples of Podostemaceae collected between 1998 and 2007 from Brazil, Costa Rica, Suriname, and Venezuela for arthropods even though these samples were not collected with the intent to investigate arthropod-Podostemaceae associations. We examined 15 samples of Podostemaceae, including 10 species never evaluated for arthropod associations, and found over 9000 arthropods representing 12 different orders. The most abundant orders were Diptera (77.88%), Trichoptera (12.90%), Coleoptera (3.35%), and Lepidoptera (2.42%). We found several arthropods not previously reported from Podostemaceae, including Collembola and Acari, documented several instances of insects boring into plant tissues, and provide the first report of an insect-induced gall on *Ceratolacispedunculatum* C.T. Philbrick, Novelo & Irgang.

## ﻿Introduction

**Figure 1.**
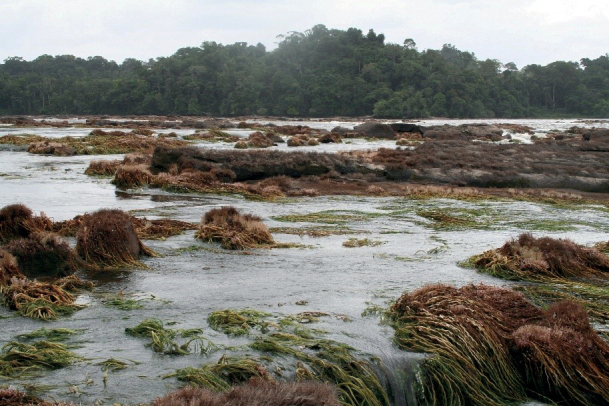
Carrao River, Orinoco River Basin, Venezuela with Podostemaceae beds.

Many aquatic arthropods have close associations with aquatic plants (Hutchens et al. 2004). One unusual family of aquatic plants with infrequently studied arthropod associations is Podostemaceae (Fig. 1). Podostemaceae (Malpighiales) comprise 50 genera and 300 species (Philbrick and Novelo 2004; Xi et al. 2012). They have distinctive morphological traits and resemble seaweeds and bryophytes (Cook and Rutishauser 2007). Their geographic distribution includes southern Asia, Africa, and the Americas, with 60% of all species found in the Americas and only one species, *Podostemumceratophyllum* Michx., found in North America (Philbrick and Crow 1983; Philbrick and Novelo 2004). These plants are also primarily found in river rapids and waterfalls attached to rocks (Philbrick and Novelo 2004; de Sá-Haiad et al. 2010).

Podostemaceae provide habitat and food for a diverse assemblage of aquatic arthropods (Tavares et al. 1998). Podostemaceae grow abundantly and form large beds that provide arthropods with protected habitat in areas with rich oxygen flow (Tavares et al. 1998). The large Podostemaceae beds and associated surface area of plant tissue can provide habitat to which arthropods can attach and feed. The plants simultaneously protect arthropods from predators and rapid water flow (Hutchens et al. 2004; Argentina et al. 2010). Arthropods commonly associated with Podostemaceae in South America include Diptera, Ephemeroptera, and Lepidoptera (Tavares et al. 1998). Similar associations have been found in North America, with a notable lack of Lepidoptera (Grubaugh and Wallace 1995). Arthropods utilize the entire plant, and chironomid larvae have been found inside the stem, inducing galls on *Marathrumutile* Tul. and *Noveloacoulteriana* (Tul.) C.T. Philbrick (Jäger-Zürn et al. 2013).

Investigating the community of arthropods that are associated with Podostemaceae is important for understanding lotic arthropod biodiversity and ecosystem conservation. Freshwater systems are extremely fragile and are easily impacted by external factors (Su et al. 2021). Some examples of human behavior that can impact these systems include dam building, introducing invasive species, and modifying the land (Su et al. 2021). The potential impact of Podostemaceae loss on aquatic biodiversity was documented in a study that experimentally removed Podostemaceae from an ecosystem (Hutchens et al. 2004). When Podostemaceae were removed, there was a 90% decrease in biomass and an 88% decrease of invertebrate species abundance (Hutchens et al. 2004). A decrease of total invertebrate biomass and species abundance can be detrimental to other species in the ecosystem because of their diverse interactions as detritivores, predators, and prey. A decrease in Podostemaceae biomass was demonstrated to decrease local fish populations because the large populations of arthropods were no longer readily available to eat for the insectivorous fish (Argentina et al. 2010).

Tavares et al. (1998) investigated insects on nine species of Podostemaceae in the genera *Apinagia*, *Mourera*, and *Rhyncholacis* in eastern Amazonia, Brazil. Their work is currently the most extensive study of arthropod-Podostemaceae associations in South America. They identified 26 families of arthropods in nine orders. Most of the arthropods identified were Diptera, making up 67% of insects found on Podostemaceae, followed by Lepidoptera (14%) and Ephemeroptera (12%) (Tavares et al. 1998). Tavares et al. (1998) were the first to document the extent of arthropod biodiversity associated with Podostemaceae in South America and they reiterated these associations in subsequent publications (Collart et al. 1998; Tavares et al. 2006).

Another study suggesting arthropod associations with Podostemaceae was in Suriname as part of an effort to expand taxonomic knowledge of aquatic arthropods in the country (Pereira and Berrenstein 2006). Although the study did not focus on arthropods on Podostemaceae, Podostemaceae were present in most sampling locations suggesting a possible baseline for understanding specific associations. The survey investigated arthropod populations in the Central Suriname Nature Reserve and found 31 families in nine different orders. Diptera were most abundant (60%), followed by Ephemeroptera (31%), and Lepidoptera (1%) (Pereira and Berrenstein 2006).

More recently, an investigation of larval Lepidoptera collected from Podostemaceae in Central and South America found several new species (Bethin et al. 2021). This study noted several species with new morphologies ranging from new respiratory structures to morphological traits with unknown purposes, such as a membranous sac and a small tail. These newly reported insect morphological traits demonstrated the potential to discover new insect diversity associated with Podostemaceae.

The urgency to understand global biodiversity has become heightened with recent reports of a 68% decline in wildlife populations between 1970 and 2016 (Almond et al. 2020). Rivers are at an especially high risk of ecosystem change with global increases in dam construction (Dethier et al. 2022). Our study sought to further document lotic arthropod biodiversity by exploring associations with Podostemaceae in Central and South America from 12 Podostemaceae species, 10 of which had never been evaluated for arthropod diversity. We chose samples collected from northern Costa Rica to southern Brazil, expanding the geographic range of known arthropod associations with Podostemaceae in Central and South America.

## ﻿Materials and methods

**Figure 2.**
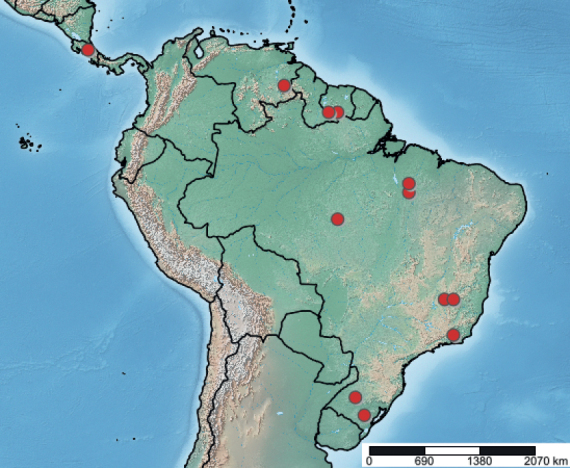
Map of sampling locations, appearing as red dots, across Central and South America (SimpleMappr). Some samples of different species were collected in the same river at the same approximate coordinates and appear as the same dot.

Podostemaceae were collected during the dry seasons in Brazil, Costa Rica, Suriname, and Venezuela for systematic botanical study (Fig. 2, Table 1). Because the original goal of the expeditions was to better understand Podostemaceae diversity, arthropod collections were incidental, and a specific collecting protocol for arthropods was not followed. Data on the locations, other than coordinates, were also not collected because they were not required for the original intent of the collections. Plant samples were collected by separating the plant from the substrate and vigorously shaking the specimen in the water to remove arthropods and debris. Individual plant specimens were then placed in separate plastic bags with 70% ethanol. The ethanol was periodically changed and emptied throughout the days of collection before being transported for study at Western Connecticut State University in the United States. The samples were transferred to plastic containers with 70% ethanol for long-term storage.

**Table 1.** Voucher specimens of Podostemaceae listing taxon, country, state/district, river, primary collector, collection number, month, year and acronym (http://sweetgum.nybg.org/science/ih/) of the herbarium where the voucher is located. Super scripts are used to differentiate between replicate species/samples.

Taxon	Country	State/District	River	Primary Collector	Collection Number	Month	Year	Acronym
***Apinagiadigitata* ^1^**	Suriname	Sipaliwini	Zuid River	Philbrick	6159	Oct.	2007	MICH
***Apinagiadigitata* ^2^**	Suriname	Sipaliwini	Zuid River	Philbrick	6171	Oct.	2007	MICH
***Apinagiarichardiana* ^1^**	Suriname	Sipaliwini	Lucie River	Philbrick	6184	Oct.	2007	MICH
***Apinagiarichardiana* ^2^**	Suriname	Sipaliwini	Lucie River	Philbrick	6155	Oct.	2007	MICH
***Apinagiariedelii***	Brazil	Tocantins	Rio Lontra	Philbrick	5992	Jul.	2006	MICH
***Ceratolacispedunculatum***	Brazil	Minas Gerais	Rio Bicudo	Philbrick	5761	Jul.	2002	MICH
***Diamantina lombardii***	Brazil	Minas Gerais	Rio do Peixe	Philbrick	5783	Aug.	2002	MICH
***Lophogyne* s.1. sp.**	Brazil	Para	Rio Sao Benedito	Bove	1864	Sep.	2007	MICH
***Marathrumfoeniculaceum***	Costa Rica	Alajuela	Rio Quequer	Philbrick	5901	Mar.	2006	MICH
***Moureraelegans***	Brazil	Para	Rio Araguaia	Philbrick	5976	Jul.	2006	MICH
***Podostemummuelleri***	Brazil	Rio Grande do Sul	Arroio do Lajeado	Philbrick	5024	Jan.	1998	MICH
***Podostemumrutifolium***	Brazil	Rio Grande do Sul	Tributary of Rio Jaguari	Philbrick	5032	Jan.	1998	MICH
***Podostemumweddellianum***	Brazil	Rio de Janeiro	Rio da Cidade	Philbrick	5000	Jan.	1998	MICH
***Rhyncholacis* sp.^1^**	Venezuela	Bolivar	Rio Carrao	Philbrick	6058	Jan.	2007	MICH
***Rhyncholacis* sp.^2^**	Venezuela	Bolivar	Rio Carrao	Philbrick	6052	Jan.	2007	MICH

We initially evaluated approximately 200 plant samples for presence of arthropods and created a catalog of approximately 100 samples with relatively higher arthropod abundance. Subsequently, we chose 15 samples to evaluate based on the presence of unique arthropods, broad geographic representation, and plant species that had not been previously investigated. The 15 Podostemaceae samples consisted of eight genera and 12 species and were collected between 1998 and 2007 from Suriname, Brazil, Costa Rica, and Venezuela (Table 1). We also assigned each sample of the same species a superscript identifier (ex: *A.richardiana*^1^) to easily distinguish between the two samples.

To evaluate each sample, we removed each stem and leaf from the collection container and rinsed it with 70% ethanol to separate the arthropods from the plant into a Petri dish where they were manually sorted and counted. We inspected each portion of the plant with a dissecting microscope at a maximum of 40× magnification to remove any remaining arthropods manually. We identified each arthropod to order and placed them in individual vials, or vials with 10–100 arthropods of the same order or family, containing 70% ethanol. After cataloging all arthropods from the samples, we calculated the proportion of each taxon in each sample. We also calculated the Shannon-Wiener diversity index for arthropods collected from each plant species as a relative indicator of arthropod diversity by plant species.

## ﻿Results

In total we counted 9197 arthropods and identified 12 orders (Table 2). Overall, Diptera were the most abundant, comprising 77.88% of all arthropods collected. Trichoptera (12.90%), Coleoptera (3.35%), Lepidoptera (2.42%), and Ephemeroptera (1.71%) were the next most abundant. The other nine orders comprised the remaining 3.45% of collected arthropods (Table 2).

**Table 2.** Proportion of arthropods found in all samples by insect order with total number of arthropod individuals counted.

Arthropod order	Total
**Acari**	0.21%
**Coleoptera**	3.35%
**Collembola**	0.01%
**Diptera**	77.88%
**Ephemeroptera**	1.71%
**Hemiptera**	0.41%
**Hymenoptera**	0.03%
**Lepidoptera**	2.42%
**Megaloptera**	0.02%
**Odonata**	0.04%
**Plecoptera**	0.5%
**Trichoptera**	12.90%
**Unknown**	0.51%
**Number of arthropods**	**9197**

Diptera were found in all 15 (100%) samples, Ephemeroptera found in 14 (93.3%), and Lepidoptera and Trichoptera were found in 13 (86.7%) samples (Table 3). *Apinagiariedelii* (Bong.) Tul. was among the most diverse samples with specimens from all 10 orders of aquatic arthropods, plus Hymenoptera (Table 3). *Moureraelegans* Baill was among the least diverse plant samples evaluated with only three arthropod orders present. However, over 1000 arthropods, mostly Diptera and Trichoptera (Table 3), were found in *Moureraelegans* making it the sample with the third highest number of arthropods in this study. The least common arthropods, found on *A.riedelii*, were Collembola (one specimen) and Megaloptera (two specimens).

**Table 3.** Proportion and total number of arthropod specimens for each order, and the Shannon-Weiner diversity index score of each sample of Podostemaceae.

	*A.digitata* ^1^	*A.digitata* ^2^	*A.richardiana* ^1^	*A.richardiana* ^2^	*A.riedelii*	*C.pedunculatum*	*D.lombardii*	*Lophogyne* s.1. sp.	*Ma.foeniculaceum*	*Mo.elegans*	*P.muelleri*	*P.rutifolium*	*P.weddellianum*	*Rhyncholacis* sp.^1^	*Rhyncholacis* sp.^2^
**Acari**	0.001	0.0006	0.034	-	-	0.006	0.010	-	-	-	0.015	-	0.003	0.100	0.038
**Coleoptera**	0.009	0.0006	-	-	0.076	0.111	0.207	-	0.082	-	0.403	0.514	0.040	-	0.038
**Collembola**	-	-	-	-	0.001	-	-	-	-	-	-	-	-	-	-
**Diptera**	0.928	0.9628	0.759	0.714	0.604	0.718	0.617	0.405	0.056	0.502	0.388	0.135	0.850	0.500	0.423
**Ephemeroptera**	0.028	0.0036	0.095	0.029	0.012	0.108	0.024	0.143	0.007	-	0.015	0.027	0.037	0.050	0.038
**Hemiptera**	-	0.0003	-	0.057	0.015	-	0.007	-	0.041	-	-	-	-	-	-
**Hymenoptera**	-	-	-	-	0.001	-	0.005	-	-	-	-	-	-	-	-
**Lepidoptera**	0.017	0.0281	0.017	0.029	0.037	0.024	-	0.190	0.020	0.005	0.015	0.243	-	0.200	0.462
**Megaloptera**	-	-	-	-	0.002	-	-	-	-	-	-	-	-	-	-
**Odonata**	-	-	0.009	-	0.001	0.003	-	-	-	-	-	-	0.003	-	-
**Plecoptera**	-	0.0032	-	0.029	0.034	-	0.002	0.024	0.002	-	-	-	-	-	-
**Trichoptera**	0.015	0.0008	0.052	0.057	0.200	0.021	0.105	0.238	0.790	0.493	0.134	0.081	0.059	-	-
**Unknown**	0.002	-	0.034	0.086	0.017	0.009	0.024	-	0.002	-	0.030	-	0.008	0.150	-
**Total arthropods**	**1721**	**3632**	**116**	**35**	**894**	**333**	**420**	**42**	**461**	**1039**	**67**	**37**	**354**	**20**	**26**
**Diversity index**	0.37	0.19	0.92	1.08	1.28	0.98	1.15	1.39	0.82	0.72	1.29	1.26	0.63	1.33	1.09

We also documented a few instances of arthropods directly using the plants. We found Diptera inside the tissue of *A.digitata* P. Royen, *A.richardiana* (Tul.) P. Royen, and *C.pedunculatum* C.T. Philbrick, Novelo & Irgang and Lepidoptera using the plant by creating a pupal case out of leaves.

The Shannon-Wiener index varied from a low index of 0.19 from *A.digitata^2^* to a high index of 1.39 from *Lophogyne* s.1. sp. (Table 3). The next two samples with the highest indices were *Rhyncholacis* sp.^1^ at 1.33 and *Podostemummuelleri* Warm at 1.29 (Table 3).

## ﻿Discussion

Our study expands the understanding of arthropods associated with Podostemaceae both geographically and taxonomically. All arthropods we identified represent an association with Podostemaceae, however, because the sampling method attempted to remove arthropods from the samples, it is likely there are additional associations. We found similar orders with similar proportional abundance as previous South American studies and added reports of arthropods on Podostemaceae species that were previously never reported (Tavares et al. 1998; Pereira and Berrenstein 2006). We increased the total Podostemaceae species evaluated for arthropods in South America from nine to 20 species. We also report the first evaluation of arthropods on *Ceratolacis* sp., *Lophogyne* sp. and *Podostemum* spp., other than *P.ceratophyllum* in North America. The arthropods found on *C.pedunculatum* and *Diamantinalombardii* Novelo, C.T. Philbrick & Irgang were part of the collections that aided in the type description of these plant species and offer a small glimpse into the potential ecological importance of these relatively new plant species. *Ceratolacis* is a ditypic genus, while *Diamantina* is monotypic, endemic to Minas Gerais, Brazil (Philbrick and Novelo 2004), highlighting the importance of understanding these unique plants.

Of the 9197 arthropods found in the samples, Diptera were dominant, comprising 77.88% of all arthropods (Table 2). However, on *P.rutifolium* and *P.muelleri*, Coleoptera were dominant (Table 3). It is not surprising that Diptera were abundant because they commonly comprise most arthropods in aquatic arthropod samples (Tavares et al. 1998; Zequi et al. 2019). We found relatively high abundance of Trichoptera and Lepidoptera, with a higher proportion of Trichoptera than previously reported on Podostemaceae (Tavares et al. 1998).

We also report the first specimen of Collembola (Entomobryomorpha) from Podostemaceae. Although it was only a single specimen, it had not been described by both Tavares et al. (1998) or Pereira and Berrenstein (2006). We also documented several mites (Acari) and it is possible the mites were parasitizing the arthropods or Podostemaceae. This likely association is especially interesting because they had not been previously noted or documented on Podostemaceae. We also documented Ephydridae on species of *Apinagia*, *Ceratolacis*, *Marathrum*, *Podostemum* and *Rhyncholacis* where it had only previously been documented on *Rhyncholacis* sp. (Tavares et al. 1998). We also found Helicopsychidae in the sample of *C.pedunculatum* where it had only been previously reported on *Apinagiaguyanenesis* (Pulle) P. Royen (Tavares et al. 1998).

We documented several arthropods directly using the plants. We found 438 Simuliidae (Diptera) pupae attached to most of the plants except *A.richardiana*, *D.lombardii*, *Lophogyne* s.1. sp., *Rhyncholacis* sp.^1^. We also documented Chironomidae (Diptera) boring into the plant tissue of *A.richardiana*^2^, *A.digitata^2^*, and *C.pedunculatum*. We are also the first to document a chironomid-induced gall on *C.pedunculatum*. We found Lepidoptera pupae that had created a casing using the leaves of the plants (Bethin et al. 2021). These documented instances of direct use by insects are likely a conservative representation of the interactions because many of the insects were removed during the collection process and in storage.

The samples with the highest diversity were *Lophogyne* s.1. sp., *Rhyncholacis* sp.^1^, *P.muelleri*, and *A.riedelii* with Shannon-Wiener values between 1.39 and 1.28 (Table 3). *Lophogyne* s.1. sp. and *Rhyncholacis* sp.^1^ had the highest diversity index values, which reflects the relatively even abundance of the orders found, even though overall abundance was low. The sample of *A.riedelii* was the most diverse, with the most orders found in a single sample, which is reflected in the higher index value. The two lowest indices, 0.19 and 0.37, were from *A.digitata*, which also contained the most arthropods with approximately 5400 arthropods combined. However, both samples were mostly Diptera, over 92%, resulting in a lower diversity index value.

Identifying arthropod-Podostemaceae associations was not the original intent of these plant collections. The incidental arthropod collections reported in this study are likely a snapshot into arthropod-Podostemaceae interactions. While the associations we identified do not represent a complete understanding because arthropods were removed during collection, they still present associations that have not been previously identified from these plant species in these locations. Simply because we examined arthropods from plants that few others have researched, we documented two new arthropod orders associated with Podostemaceae and added 10 Podostemaceae species to the total evaluated for arthropod associations.

With this expanded knowledge of these freshwater communities, conservation and restoration efforts can reference our data as baseline evidence for the contribution of Podostemaceae to river biodiversity throughout Central and South America. It is evident that Podostemaceae create unique habitats ideal for arthropod diversity. As deforestation and dam construction continue in Central and South America, the likely impact on rivers could contribute to loss of biodiversity before it is even documented. It is clear that more research is needed to fully understand the extent of arthropod diversity found in Podostemaceae beds and the ecological importance of these communities.
